# IL-1R1 signaling in TBI: assessing chronic impacts and neuroinflammatory dynamics in a mouse model of mild closed-head injury

**DOI:** 10.1186/s12974-023-02934-3

**Published:** 2023-10-26

**Authors:** Jonathan C. Vincent, Colleen N. Garnett, James B. Watson, Emma K. Higgins, Teresa Macheda, Lydia Sanders, Kelly N. Roberts, Ryan K. Shahidehpour, Eric M. Blalock, Ning Quan, Adam D. Bachstetter

**Affiliations:** 1 Department of Neuroscience, University of Kentucky, 741 S. Limestone St., Lexington, KY 40536 USA; 2 Spinal Cord and Brain Injury Research Center, University of Kentucky, Lexington, KY USA; 3 Sanders-Brown Center on Aging, University of Kentucky, Lexington, KY USA; 4 MD/PhD Program, University of Kentucky, Lexington, KY USA; 5 Department of Cell, Developmental, and Integrative Biology, University of Alabama at Birmingham, Birmingham, AL USA; 6 Department of Pharmacology and Nutritional Sciences, University of Kentucky, Lexington, KY USA; 7 Department of Biomedical Science, Charles E. Schmidt College of Medicine and Brain Institute, Florida Atlantic University, Jupiter, FL USA

**Keywords:** Neuroinflammation, Interleukin-1, Interleukin-1 receptor-1, Astrocyte, Microglia, Traumatic brain injury

## Abstract

Neuroinflammation contributes to secondary injury cascades following traumatic brain injury (TBI), with alternating waves of inflammation and resolution. Interleukin-1 (IL-1), a critical neuroinflammatory mediator originating from brain endothelial cells, microglia, astrocytes, and peripheral immune cells, is acutely overexpressed after TBI, propagating secondary injury and tissue damage. IL-1 affects blood–brain barrier permeability, immune cell activation, and neural plasticity. Despite the complexity of cytokine signaling post-TBI, we hypothesize that IL-1 signaling specifically regulates neuroinflammatory response components. Using a closed-head injury (CHI) TBI model, we investigated IL-1's role in the neuroinflammatory cascade with a new global knock-out (gKO) mouse model of the IL-1 receptor (IL-1R1), which efficiently eliminates all IL-1 signaling. We found that IL-1R1 gKO attenuated behavioral impairments 14 weeks post-injury and reduced reactive microglia and astrocyte staining in the neocortex, corpus callosum, and hippocampus. We then examined whether IL-1R1 loss altered acute neuroinflammatory dynamics, measuring gene expression changes in the neocortex at 3, 9, 24, and 72 h post-CHI using the NanoString Neuroinflammatory panel. Of 757 analyzed genes, IL-1R1 signaling showed temporal specificity in neuroinflammatory gene regulation, with major effects at 9 h post-CHI. IL-1R1 signaling specifically affected astrocyte-related genes, selectively upregulating chemokines like Ccl2, Ccl3, and Ccl4, while having limited impact on cytokine regulation, such as Tnfα. This study provides further insight into IL-1R1 function in amplifying the neuroinflammatory cascade following CHI in mice and demonstrates that suppression of IL-1R1 signaling offers long-term protective effects on brain health.

**Supplementary Information:**

The online version contains supplementary material available at 10.1186/s12974-023-02934-3.

## Introduction

Traumatic brain injury (TBI) is a public health problem that impacts individuals of all ages and demographics. The types of biomechanical forces exerted on the brain can differ dramatically in TBIs caused by assault, falls, blast injuries, sport-related concussions, or motor vehicle-related accidents. This results in differing severity and pathological locations of the primary injury. Following the primary TBI-related injury, a chain reaction of intricate pathophysiological events occurs, recognized as a secondary injury that, when it persists without appropriate intervention, may contribute to post-concussion syndrome, long-term disability, and potential neurodegeneration [1–3].

Numerous studies have highlighted TBI-induced inflammation in the brain as a critical progenitor of the secondary pathophysiological progression of TBI [4, 5]. Research in animal models and human TBI patients demonstrates that the neuroinflammatory reaction to a TBI occurs in activating and resolving waves of different inflammatory mediators, highlighting the importance of their magnitude and temporal dynamics [5–7]. Despite the rapid release of DAMPs, chemokines, and cytokines following TBI, we hypothesize that IL-1 signaling exhibits specificity in propagating TBI-induced neuroinflammation. IL-1 plays a central role in physiological and pathological processes throughout the body and central nervous system (CNS), including autoimmune diseases, sleep regulation, mood disorders, memory consolidation, psychological stress, and neurodegenerative diseases [8–12]. Previous studies have also demonstrated a significant elevation of IL-1 in the brain post-TBI [13]. Importantly, the primary receptor for IL-1, IL-1 receptor-1 (IL-1R1), is found in brain endothelial cells, neurons, and astrocytes, making IL-1/IL-1R1 signaling temporally and spatially appropriate to propagate neuroinflammation [14–18].

Many studies using biomechanically unique models of TBI, completed over the last two decades, have demonstrated that manipulating the IL-1/IL-1R1 pathway with pharmacological or genetic approaches protects brain health and reduces neuroinflammation [19–34]. However, clinical interventions have not yet been tested, and the effects of inhibiting IL-1 signaling on the healing process are still unclear. Moreover, previous studies used an IL-1R1 KO mouse model with incomplete loss of IL-1 receptor signaling in the brain [18]. In these models, residual IL-1R1 expression persisted through various receptor isoforms. Among these, a distinctive one, IL-1R3, primarily located in neural tissues, does not signal through NF-κB and p38 MAPK. Instead, it interacts with IL-1RAcPb to induce an increase in Kv current via the Akt kinase. These prior models could not definitively isolate the effects of IL-1R1 signaling due to the partial loss-of-function nature of these KO mice.

In contrast, our study utilizes a newly developed global *IL-1R1 KO* (gKO) mouse model that more effectively eliminates IL-1 signaling in the brain and periphery [14]. This gKO model provides a more thorough and unequivocal approach to investigating the role of IL-1/IL-1R1 signaling, making it a significant improvement over the traditional IL-1R1 KO model. The use of the gKO model in our research is indeed a key advantage as it allows for a more definitive exploration of the impact of a complete lack of IL-1 signaling, providing a more precise understanding of IL-1R1's role in the pathogenesis of TBI.

In light of these advancements, we investigated the consequences of losing IL-1R1 signaling in a mouse model of mild closed-head injury (CHI) 14–16 weeks after injury. We found a sustained decrease in behavioral deficits and gliosis. We then set out to determine if IL-1R1 was instrumental during the acute inflammatory response, influencing both the propagation and resolution of inflammation. Our findings indicate that other inflammatory mediators cannot compensate for the loss of IL-1R1 and that the 9-h window is crucial for IL-1R1's maximal effects on neuroinflammatory signaling.

## Methods

### Animals

This research was conducted in compliance with the NIH’s Guide for the Care and Use of Laboratory Animals and was approved by the University of Kentucky’s Institutional Animal Care and Use Committee. All mice in this study were male and age-matched. Only males were used due to the availability of the mice at the time and budget constraints. All experiments used 5-month-old male mice (WT = 5.07 ± 0.79 months, KO = 5.17 ± 0.55 months; mean ± SD). IL-1R1 gKO mice (C57BL/6N-Il1r1^tm1Quan^) [14] mice obtained in collaboration with Ning Quan Lab and an independent colony was maintained at the University of Kentucky. The IL-1R1 gKO mice were crossed to a C57BL/6J for more than 4 generations. WT littermates of the gKO mice or C57BL/6J mice obtained from Jackson Laboratory were randomly assigned to control groups. The genotyping of the mice for the IL-1R1 gene was confirmed by Transnetyx, Inc (Cordova, TN, USA).

### Closed-head injury model

Mice of each genotype (WT and IL-1R1 gKO) were randomly assigned to receive either a closed-head injury (CHI) or sham procedure as previously described [35, 36]. The animals were anesthetized with 2.5% isoflurane before having their heads shaved. The mice assigned to receive the CHI were administered continuous 2.5% isoflurane with a non-rebreathing nasal cone attached to a passive exhaust system. Their heads were stabilized using non-traumatic ear bars fixed to a stereotaxic frame (Stoelting Co., Wood Dale, USA). A 1-mL latex pipette bulb was filled with water and positioned under each mouse's head to displace the impact force. This apparatus was constructed overtop a heating pad set to 37 °C to keep the mice’s temperatures stable. Eye ointment was also used, and betadine solution was used to disinfect the scalps. The skull was made visible by a midline incision in the scalp, and a 5.0-mm steel tip impounder was used to induce the single controlled impact at coordinates: ML = 0.0 mm; AP = − 1.5 mm, with a velocity of 5.0 ± 0.2 m/s and impact depth of 1.0 mm. Mice in the sham-procedure group underwent identical pre-injury procedures but with no impact delivered. Following surgery, scalps were closed using surgical staples, and the mice were transferred to a recovery cage, where they were continuously monitored until fully ambulatory.

### Active avoidance

Behavioral characterization of the mice was assessed using the active avoidance test. This procedure assesses the ability of the mice to learn to avoid an aversive stimulus by responding to a warning signal. To perform this test, we employed a modified protocol of Macheda et al. [35] using a Gemini shuttle box. The mice were tested for five consecutive days with 50 trials per day. During the test, mice were gently placed in one of the two dark compartments of the shuttle box and allowed to explore both sides for 300 s. After the habituation period, a house light (CS) was presented for 10 s in the empty compartment, followed by a 0.2 mA foot shock (US) delivered for 2 s. The mouse could "avoid" the shock by crossing to the opposite compartment while the warning signal (CS) was on. Alternatively, the mouse could "escape" by crossing to the opposite compartment during the shock delivery. If the subject remained in the original compartment and received the shock, a "no response" was registered. Once the mouse crossed to the opposite compartment, both the CS and US signals ceased, and an intertrial interval (ITI) began. During the ITI, which lasted 30 ± 5 s, the mouse could freely move between the two sides of the shuttle box. A new trial started automatically after the ITI period had elapsed. The only deviation from the original protocol [35] was that we began counting the 50 trials on day one only after an escape or a no-response trial was recorded. The Gemini software (San Diego Instruments) recorded the number of times the animal crossed between chambers, avoided, escaped, and failed trials, as well as the shuttle time between the two compartments after the CS was presented. We analyzed the percentage of avoided trials and the latency to cross to the safe compartment.

### Euthanasia and brain tissue collection

Mice were deeply anesthetized with 5% isoflurane before being subjected to transcranial perfusion using ice-cold 1 × PBS for 5 min. Their brains were rapidly removed and dissected. The right hemisphere was further dissected, flash-frozen in liquid nitrogen, and stored at − 80 °C for subsequent biochemical analysis. Meanwhile, the left hemisphere was fixed in 4% paraformaldehyde overnight and then cryoprotected using a 30% sucrose/PBS solution.

### Immunohistochemistry

Coronal slices (30 μm) from the left-brain hemisphere were prepared using a sliding microtome with a freezing stage and preserved in cryoprotectant at − 20 °C. Staining was performed on free-floating sections, selecting every 10th section from the left cerebral cortex between approximately 1.2 mm and − 3 mm from the bregma. Primary and secondary antibodies were mixed in 3% normal goat serum (NGS) (Lampire Biological Laboratories, catalog no. 7332500) containing 0.2% Triton X-100. Endogenous peroxidase activity was quenched with 3% H2O2 in methanol. The tissue was then blocked with 10% NGS containing 0.2% Triton X-100 for 1 h before the primary antibody was applied for 14–16 h. Detection of the primary antibody was achieved using a biotinylated secondary antibody (1:500, Biotin Goat Anti-Rabbit Vector Laboratories BA-1000 RRID:AB_2313606), and the signal was amplified using an avidin–biotin substrate (ABC solution, Vector Laboratories catalog no. PK-6100), with color development in 3,3′-diaminobenzidine tetra-hydrochloride (DAB; Sigma, catalog no. D5637). Glial activation markers included rabbit anti-GFAP (1:10,000 Dako, catalog no. Z0334 RRID:AB_10013382) for astrocytes and rabbit anti-IBA1 (1:10,000, Wako, catalog no. 019-19741) for microglia.

### Quantitative image analysis of immunohistochemical stains

The entire stained slide was imaged at 20× magnification using the Zeiss AxioScan Z.1 digital slide scanner to produce a high-resolution digital image. The neocortex and hippocampus regions were outlined with the HALO imaging software (RRID:SCR_018350). The HALO area fraction algorithm quantified specific staining in the region, with the number of positive pixels normalized to the area outlined to account for regional size variations. All slides in the batch were analyzed using the same parameters. The color markup analysis was confirmed for each slide. A blinded observer conducted all quantifications.

### RNA isolation

RNA from the cortex under the injury was isolated using the Qiagen QI Shredder columns (Qiagen, catalog no. 79656) and RNeasy Plus Minikit according to the manufacturer’s instructions (Qiagen, catalog no. 74136). RNA quantity and quality were determined using A260/A280 readings by NanoDrop (Thermo Scientific). RNA was then diluted to 30 ng/µl.

### Nanostring nCounter gene expression analysis

RNA integrity number (RIN) was determined using Agilent 2100 Bioanalyzer and was between 9.5 and 10 for all samples. In the nCounter® assay (NanoString® Technologies, Seattle, WA), 150 ng (5 µl of 30 ng/µl) of RNA isolated and extracted as described above was assessed using the mouse-specific nCounter® Neuroinflammatory panel. Samples were prepared using a nCounter® Prep Station, and RNA complexes were immobilized on nCounter® cartridges for data collection; data were collected on a NanoString Digital Analyzer.

### Statistical analysis

Statistical analysis was conducted using JMP Pro software version 17.0 (SAS Institute, Cary, NC, USA). GraphPad Prism version 9 (GraphPad Software, San Diego, CA) was used for data visualization.

The active avoidance data were analyzed using a standard least square repeated-measures model, which accounted for the day of testing, the independent variable being examined, and the interaction between them. The results were reported as the percentage of avoided trials and least square means. Differences between mean were considered significant at *α* = 0.05.

To account for batch effects in the IHC, data were normalized to the percentage of sham for each staining batch. All staining batches included an equal representation of all experimental groups. Histochemical staining was analyzed using a two-way ANOVA, considering injury and genotype as factors. If a significant effect of injury or injury-by-genotype interaction was found, a contrast test was conducted to evaluate differences in the following groups: WT + sham vs. WT + CHI, gKO + sham vs. gKO + CHI, and WT + CHI vs. gKO + CHI. Differences between mean were considered significant at *α* = 0.05.

NanoString gene expression count data were assessed for quality control using the NanoString nCounter software platform by comparing count data to 10 positive control housekeeping genes. The data were then exported to JMP Software version 16.0 (SAS Institute, Cary, NC) for further statistical analysis. In JMP, the data were normalized and centered using geometric means, and the *Z*-score of each gene was calculated. An FDR-corrected *p*-value of < 0.05 was used to determine the significance of gene expression. Effect sizes (*Z*-scores) greater than 0 indicated an increase, and less than 0 a decrease, relative to sham control. The total significant differentially expressed genes per NanoString Neuroinflammatory pathway group were calculated for pathway enrichment analyses.

To evaluate the overexpression of significant differentially expressed genes within each NanoString Neuroinflammatory pathway, a binomial approach was employed to compare the proportion of significant differentially expressed genes in each pathway to the proportion of total significant differentially expressed genes overall. A two-way ANOVA was performed for representative gene expression data to evaluate the effect of injury (sham, 3 h, 9 h, 24 h, 72 h), genotype (WT, KO), and interaction of these two factors. Post hoc tests were used to compare those genes with a significant effect in the two-way ANOVA. A Dunnett’s multiple comparisons test compared 3 h, 9 h, 24 h, and 72 h to the sham control group. An unpaired two-tailed *t*-test was used to compare the effect of genotype at each time point. Differences between means were considered significant at *α* = 0.05.

## Results

### Loss of IL-1R1 signaling decreases reactive microglia in the neocortex and reactive astrocytes in the corpus callosum and hippocampus, and attenuates behavioral impairment 14 weeks after single CHI

**Fig. 1:**
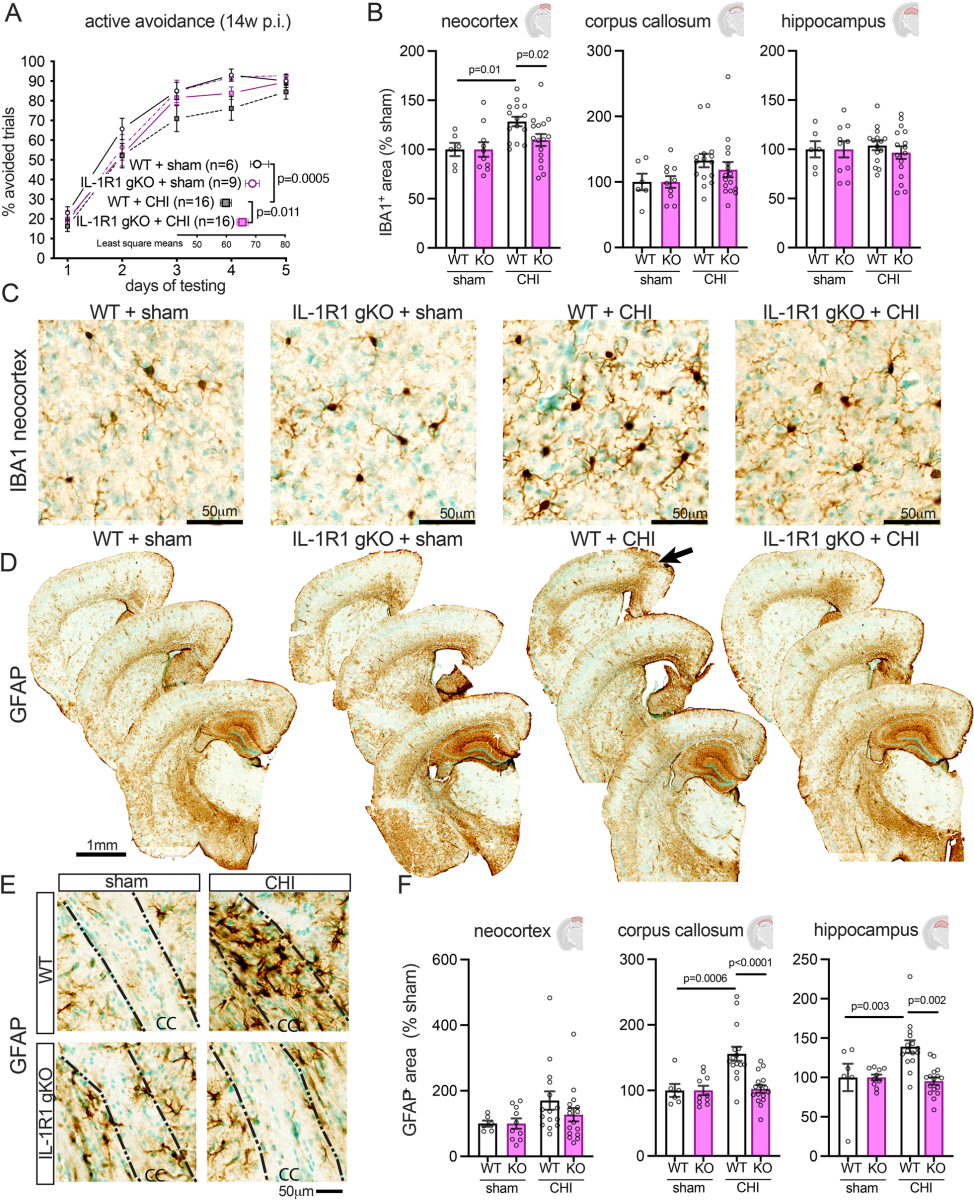
Long-term behavioral and histological effects of single mild closed-head injury in WT and IL-1R1 gKO mice. Wild-type (WT) and interleukin-1 receptor 1 global knock-out (gKO) mice underwent either a sham procedure or a closed-head injury (CHI). **A** Behavioral responses were assessed using the active avoidance method at 14 weeks post-injury. IL-1R1 gKO mice displayed enhanced behavioral function post-CHI compared to WT + CHI mice. **B** IBA1 + staining quantification in the neocortex revealed fewer injury-induced changes in gKO mice than in WT + CHI. No effects of injury or genotype were observed in the corpus callosum or hippocampus at 16 weeks post-injury. **C** Sample images of IBA1 in the neocortex. Neocortical changes in GFAP staining are marked by the arrow in **D**, and in the corpus callosum (CC) in **E**. **F** Staining intensity was quantified across 6–9 sections for the entire brain region. Statistical analysis used a standard least squares repeated-measures model for **A** and a two-way ANOVA with a post hoc test for **B**, **F**. The significance threshold was set at *p* < 0.05. Data are shown as mean ± SEM. Sample sizes: WT + sham = 6, WT + CHI = 15–16, KO + sham = 10, KO + CHI = 16

Previous studies have shown that IL-1R1 KO mice exhibit reduced cognitive deficits in the Morris water maze at 3-months post-injury in a single CHI model [32], and at 6 months post-injury in a repetitive CHI model [34]. Our aim was to build upon these findings by using additional injury and behavioral parameters. Specifically, we subjected IL-1R1 gKO and WT mice to either a sham injury or CHI. At 14 weeks post-injury, the mice underwent behavioral testing using our established active avoidance procedure [35]. Our results indicate that the IL-1R1 gKO + CHI group learned to avoid the shock stimulus more quickly than the WT + CHI group (*p* < 0.011), while the WT + CHI group performed significantly worse than the sham group (*p* = 0.0005) (Fig. 1A). These findings suggest that by eliminating IL-1R1 signaling, the mice recovered learned behavioral function more quickly than their WT counterparts following CHI.

While the CHI model does not result in gross neuronal loss, it does lead to chronic changes in microglia and astrocytes after injury, and inflammation is linked to behavioral impairments in this CHI model [13]. Consequently, we tested the potential role of IL-1R1 signaling in regulating chronic microglia and astrocyte reactivity following a mild CHI. The brain tissue was collected 1 week after the end of the active avoidance behavior, and the tissue was sectioned and labeled with anti-IBA1 and anti-GFAP to evaluate reactive microglia and astrocyte populations in the neocortex (closest to injury), corpus callosum, and hippocampus.

We found a significant decrease in IBA1^+^ microglia in the IL-1R1 gKO + CHI group compared to the WT + CHI group in the neocortex (*p* = 0.02) (Fig. 1B, C). The WT + CHI group exhibited a significant increase in IBA1 + microglia compared to the WT + sham mice (*p* = 0.01) (Fig. 1B, C). However, no significant difference in injury or genotype effect on reactive microglia populations was observed following a mild CHI in the corpus callosum or hippocampus (Fig. 1B, C).

Our findings also reveal an impact on reactive astrocyte populations in different brain regions. Specifically, we observed a significant increase in the number of reactive astrocytes detected in the corpus callosum (*p* = 0.0006) and hippocampus (*p* = 0.003) of the WT + CHI group (Fig. 1D–F). However, the IL-1R1 gKO + CHI group demonstrated a notable decrease in reactive astrocyte populations in the corpus callosum (*p* < 0.0001) and hippocampus (*p* = 0.002) compared to the WT + CHI group (Fig. 1D–F). Our findings indicate that the IL-1R1 gKO + CHI group had a reduced reactive astrocyte response compared to the WT + CHI group, suggesting that the loss of IL-1R1 signaling may contribute to less chronic glial changes following CHI.

### Temporal changes in neuroinflammatory-related genes following a CHI in WT and IL-1R1 gKO mice

**Fig. 2:**
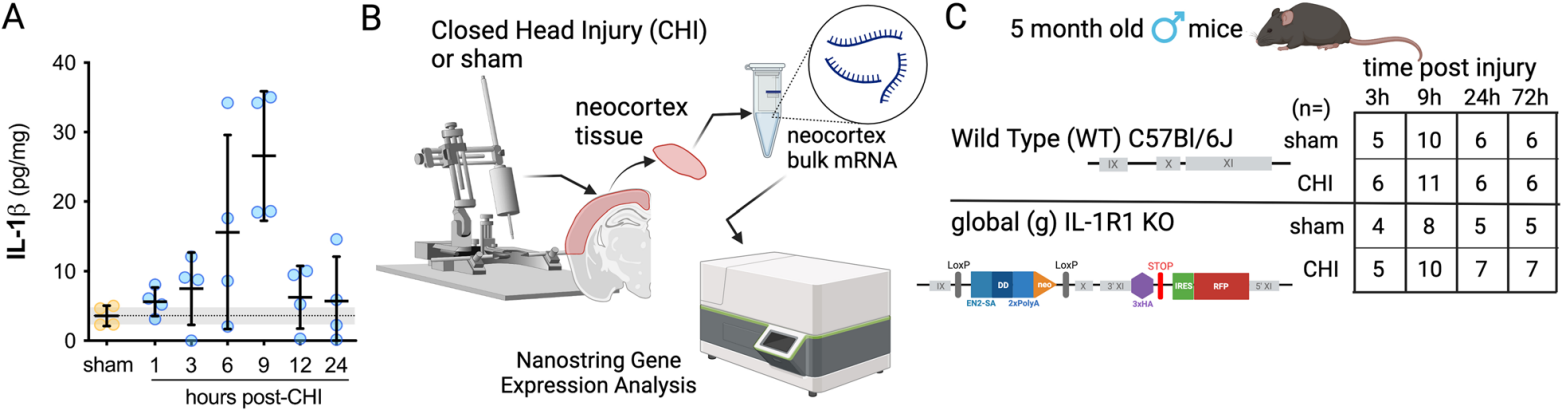
Study design. **A** Reanalysis of previously published data for Bachstetter et al. [13] demonstrates the temporal pattern of IL-1β protein changes in the injured neocortex over time. Each circle represents an individual animal. The summary statistic shows the mean and standard deviation. **B** A graphic summary of the study design shows that the neocortex tissue was collected from the injured brain. RNA was isolated from the bulk tissue, and the samples were analyzed using the NanoString mouse Neuroinflammatory panel. **C** The study used wild-type (WT) and Interleukin-1 receptor-1 global knock-out (IL-1R1 gKO) mice at 3 h, 9 h, 24 h, and 72 h post-injury. Sham-injured mice were included at each post-injury time point. The number of male mice included at each time point is shown in the table. Data are plotted as mean ± SD

After observing that IL-1R1 gKO mitigates long-term behavioral deficits and changes in reactive microglia and astrocyte following a CHI, we sought to identify whether IL-1 signaling activates any inflammatory pathways in the early stages after injury, which might explain these changes. We focused on the initial hours post-injury as we have previously reported that a CHI in young male mice causes an elevation in IL-1β protein levels in the cortex during the first 24 h, peaking at 9 h post-injury [13] (Fig. 2A). We anticipated that the peak in IL-1β protein levels would correlate with IL-1R1-dependent signaling engagement. To assess the IL-1/IL-1R1 dependent transcriptional network, we examined gene expression changes in the injured neocortex tissue (Fig. 2B) at four post-injury time points (Fig. 2C). We opted for these time points to encompass the onset of IL-1R1 elevation and peak inflammation and resolution time points (Fig. 2A).

We observed that the IL-1R1 gKO mice had a higher weight compared to the WT mice (*p* = 0.003, *t*-Test; WT = 32.11 ± 5.07 g, KO = 36.09 ± 3.60 g; mean ± SD) (Additional file 1: Fig. S1A). There was no significant difference in age (*p* = 0.614, *t*-Test; WT = 5.07 ± 0.79 months, KO = 5.17 ± 0.55 months; mean ± SD). This finding aligns with previous studies of IL-1R1 gKO mice, where larger body size was linked to increased fat mass [37]. The IL-1R1 gKO mice also exhibited shorter righting reflex times (*p* < 0.001, *t*-Test; WT = 1037.3 ± 397.4 s, KO = 417.7 ± 145.2 s; mean ± SD) (Additional file 1: Fig. S1B). Notably, the righting reflex times were strongly correlated with body weight (*p* = 0.0012, *R*^2^ = 0.32) (Additional file 1: Fig. S1C), indicating that the differences in depth/metabolism of isoflurane anesthesia associated with the increased size and fat mass of the IL-1R1 gKO mice, may contribute to the difference in righting reflex times between the groups.

**Fig. 3:**
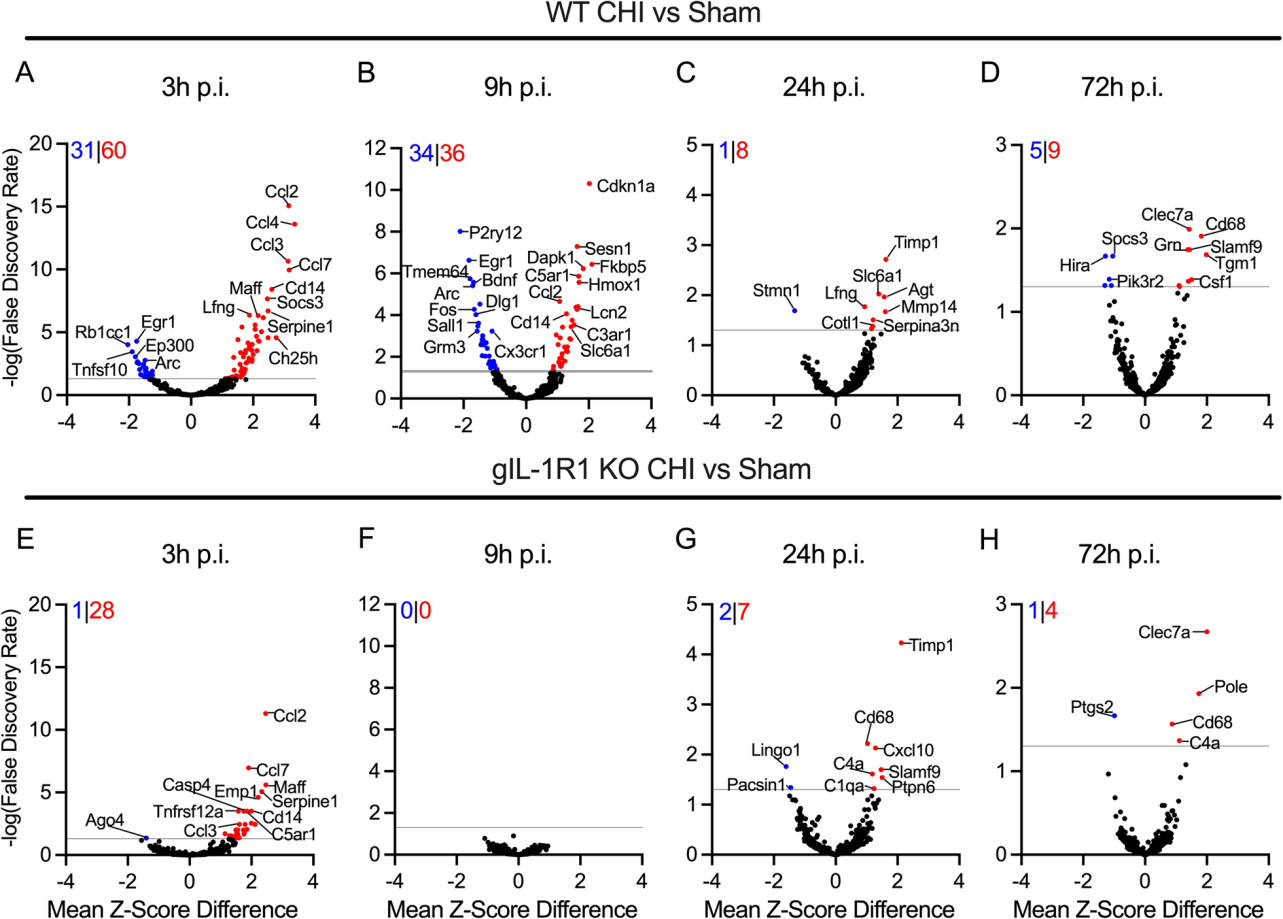
Temporal pattern of neuroinflammatory-associated gene expression caused by a CHI in WT and IL-1R1 gKO mice. Volcano plots of injury-induced differential gene expression 3 h (**A**, **E**), 9 h (**B**, **F**), 24 h (**C**, **G**), and 72 h (**D**,** H**) post-injury. Data points above the reference line (grey) represent statistical significance (FDR < 0.05) using the NanoString analysis using a mouse Neuroinflammatory panel. Positive Mean *Z*-Score Difference (red dots) indicates higher gene expression relative sham mice. Negative Mean *Z*-Score Difference (blue dots) indicates lesser gene expression relative sham mice. The top left corner of each graph displays the total number of differentially expressed genes, with downregulated genes in blue and upregulated genes in red

To understand how a CHI affected the temporal pattern of neuroinflammatory-related genes, we first evaluated in WT mice the differentially expressed genes (DEGs) between the CHI mice and sham-injured mice at each timepoint to define the injury effect on the neuroinflammatory profile. The greatest change in gene expression occurred at the 3 h post-injury timepoint (Fig. 3A). At 3 h post-injury the WT mice showed a total of 91 significant DEGs (FDR < 0.05; upregulated DEGs = 60, downregulated DEGs = 31). Chemokines (*Ccl2, Ccl3, Ccl4,* and *Ccl7*) were among the genes that were most highly upregulated in the CHI mice compared to the sham mice at 3 h post-injury (Fig. 3A).

At 9 h post-injury, the injured WT mice showed 70 total significant DEGs when compared to WT sham mice (FDR < 0.05; upregulated DEGs = 36, downregulated DEGs = 34) (Fig. 3B). Downregulated genes in the WT + CHI mice compared to sham mice include the homeostatic microglia-specific gene, *P2RY12*, the early growth response factor 1, *EGR1*, and the post-synaptic density scaffolding protein, *DLG1* (Fig. 3B)*.* Upregulated genes include cyclin dependent kinase inhibitor 1A, *CDKN1A*, sestrin 1, *SESN1*, and FKBP prolyl isomerase 5, *FKBP5* (Fig. 3B).

By 24 h post-injury, DEGs were significantly reduced compared to earlier post-injury timepoints. At 24 h post-injury, the injured WT mice showed 9 total significant DEGs when compared to WT sham mice (FDR < 0.05; upregulated DEGs = 8, downregulated DEGs = 1) (Fig. 3C)*.* Here, *TIMP1*, a protein associated with the inhibition of matrix metalloproteinases, was the most highly upregulated gene in the WT mice with injury. Stathmin 1 (*Stmn1*), a ubiquitous cytosolic phosphoprotein with the function of destabilizing microtubules, was the most downregulated gene. At 72 h post-injury, the injured WT mice showed 14 significant DEGs (FDR < 0.05; upregulated DEGs = 9, downregulated DEGs = 5) when compared to WT sham mice (Fig. 3D). The most significantly upregulated of these DEGs includes *CD68*, suggesting reactive microglia/macrophage responses are still active in repairing the injured brain. Overall, the acute phase of the neuroinflammatory response has mostly resolved by 72 h post-CHI.

Similar to the WT mice, the total number of differentially expressed genes in the IL-1R1 global knock-out (IL-1R1 gKO) mice peaked at 3 h post-injury (Fig. 3E). The IL-1R1 gKO mice showed 29 total significant DEGs (FDR < 0.05; upregulated DEGs = 28, downregulated DEGs = 1) when compared to sham. At the 3 h post-injury time point, many of the upregulated genes in the IL-1R1 gKO were the same genes upregulated at the same timepoint in the WT control mice with injury, including *Ccl2, Ccl3,* and *Ccl7*.

Surprisingly, no significant DEGs were detected at 9 h post-injury for the IL-1R1 gKO injured mice compared to IL-1R1 sham mice (Fig. 3F). The lack of DEGs in the IL-1R1 gKO mice at 9 h post-injury corresponds temporally with the peak of injury-induced IL-1β protein levels in wild-type mice (Fig. 2A). These results demonstrate a discrete temporal patterning of the neuroinflammatory response that requires IL-1/IL-1R1 signaling.

Despite no IL-1-induced neuroinflammatory response at 9 h post-injury in the IL-1R1 gKO mice, we observed a rebound in the neuroinflammatory response at 24 h post-injury (Fig. 3G), with further decrease in neuroinflammatory DEGs at 72 h post-injury (Fig. 3H). Surprisingly, at 24 h post-injury, 9 significant DEGs were identified in the IL-1R1 gKO injured mice compared to sham mice, which was equivalent to the number of DEGs seen in the WT mice at the 24 h post-injury, but with a different profile. However, by 72 h post-injury, only 5 significant DEGs were identified as a function of injury in the IL-1R1 gKO mice (Fig. 3H), including 1 downregulated DEG (*Ptgs2*) and 4 upregulated DEGs (*Clecl7a, Pole, Cd68,* and C4a).

### Pathway analysis of injury-induced changes in temporal neuroinflammatory response in WT and IL-1R1 gKO mice

**Fig. 4:**
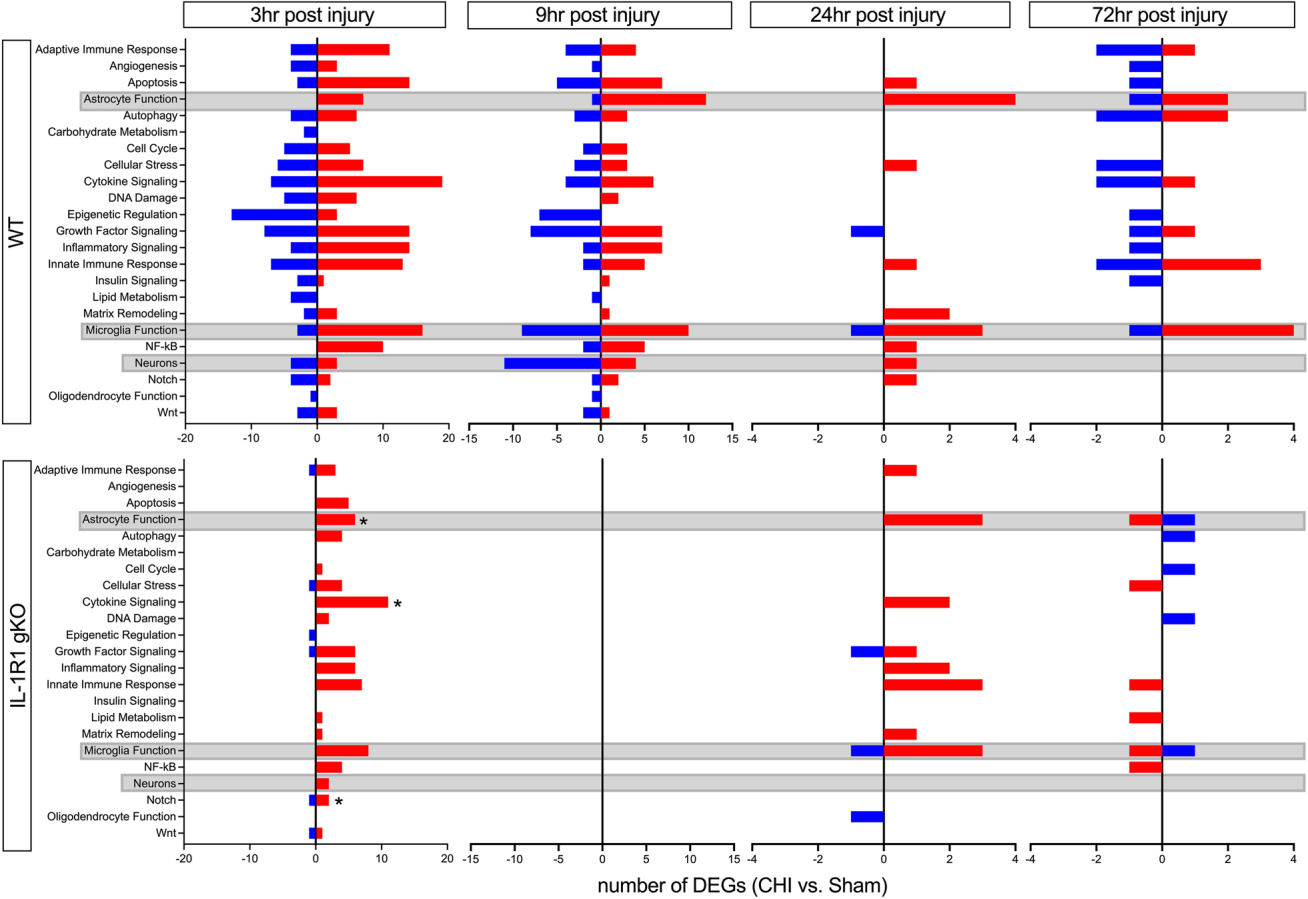
Pathway enrichment analysis of the effect of brain injury in WT and IL-1R1 gKO mice. The number of significant differentially expressed genes (DEGs) within their respective functional groups, shown as upregulated (red bars) and downregulated (blue bars), were determined based on annotations from the NanoString Neuroinflammatory panel. Genes were permitted to overlap across multiple functional groups. To evaluate whether the total number of significant DEGs within each functional category differed significantly from chance, we employed a binomial approach, calculating the probability that the total number of significant DEGs per category resulted in significant changes for that functional group (*p < 0.05). Gray boxes indicate functional gene groups of interest, including astrocyte, microglial, and neuronal function

To further understand the functional relevance of the injury effect on acute transcriptomic changes, we performed a pathway enrichment analysis comparing the significant DEGs identified in Fig. 4 to their respective functional roles using the annotations provided by the NanoString Neuroinflammatory panel, which includes 23 distinct neuroinflammatory pathways (Fig. 4). This analysis considers both the significantly upregulated and downregulated genes within their respective pathways.

The results show that the WT + CHI group had the highest number of pathway changes at 3 h post-injury, specifically in the notch signaling pathway with 26.1% (6 DEGs, 23 total annotated genes in pathway) of the genes significantly differentially expressed. This was followed by the epigenetic regulation and cytokine signaling pathways, with 22.9% (16 DEGs, 70 total annotated genes in pathway) and 22.4% (26 DEGs, 116 total annotated genes in pathway) of their respective genes being significantly differentially expressed (Fig. 4). In comparison, the IL-1R1 gKO + CHI group had the most changes in the notch signaling pathway with 13.0% (3 DEGs, 23 total annotated genes in pathway) of genes being significantly differentially expressed, followed by astrocyte function and cytokine signaling pathways with 10.9% (6 DEGs, 55 total annotated genes in pathway) and 9.5% (11 DEGs, 116 total annotated genes in pathway) of their respective genes being significantly differentially expressed.

At 9 h post-injury, the WT + CHI group had the most pathway changes in the astrocyte function pathway with 23.6% (13 DEGs, 55 total annotated genes in pathway) of the genes significantly differentially expressed. This was followed by neurons and neurotransmission, with 18.8% (15 DEGs, 80 total annotated genes in pathway) of their respective genes being significantly differentially expressed. In contrast, no genes were significantly differentially expressed in any functional pathways for the IL-1R1 gKO + CHI group (Fig. 4).

At 24 h post-injury, the WT + CHI group continued to have the most pathway changes in the astrocyte functional pathway, with 7.3% (4 DEGs, 55 total annotated genes in pathway) of the genes being significantly differentially expressed. This was followed by the matrix remodeling pathway with 4.7% (2 DEGs, 43 total annotated genes in pathway) of genes being significantly differentially expressed (Fig. 4). On the other hand, the IL-1R1 gKO + CHI group showed the most pathway changes in the astrocyte function pathway with 5.5% (3 DEGs, 55 total annotated genes in pathway) of genes being significantly differentially expressed, followed by the oligodendrocyte function group with 3.7% (1 DEG, 27 total annotated genes in pathway) of their respective genes being significantly differentially expressed.

At 72 h post-injury, the WT + CHI group continued to have the most pathway changes in the astrocyte functional pathway with 5.5% (3 DEGs, 55 total annotated genes in pathway) of genes being significantly differentially expressed, followed by the autophagy pathway with 4.0% (4 DEGs, 99 total annotated genes in pathway) of genes being significantly differentially expressed. The innate immune response pathway also had significant changes, with 3.4% (5 DEGs, 145 total annotated genes in pathway) of genes being significantly differentially expressed. In comparison, the IL-1R1 gKO + CHI group showed the most significant DEGs in the lipid metabolism pathway with 5.6% (1 DEG, 18 total annotated genes in pathway) of genes being significantly differentially expressed. This was followed by astrocyte function at 3.6% (2 DEGs, 55 total annotated genes in pathway) and Nfkβ at 1.7% (1 DEG, 59 total annotated genes in pathway) when compared to the IL-1R1 gKO + sham group (Fig. 4). These results demonstrate the differential gene expression changes occurring in various functional pathways related to neuroinflammation following CHI injury, and that IL-1 signaling unique contributions to the propagating the injury-induced inflammation.

### IL-1R1 signaling pathway reveals significant involvement in transcriptional changes 9 h after CHI in mice

**Fig. 5:**
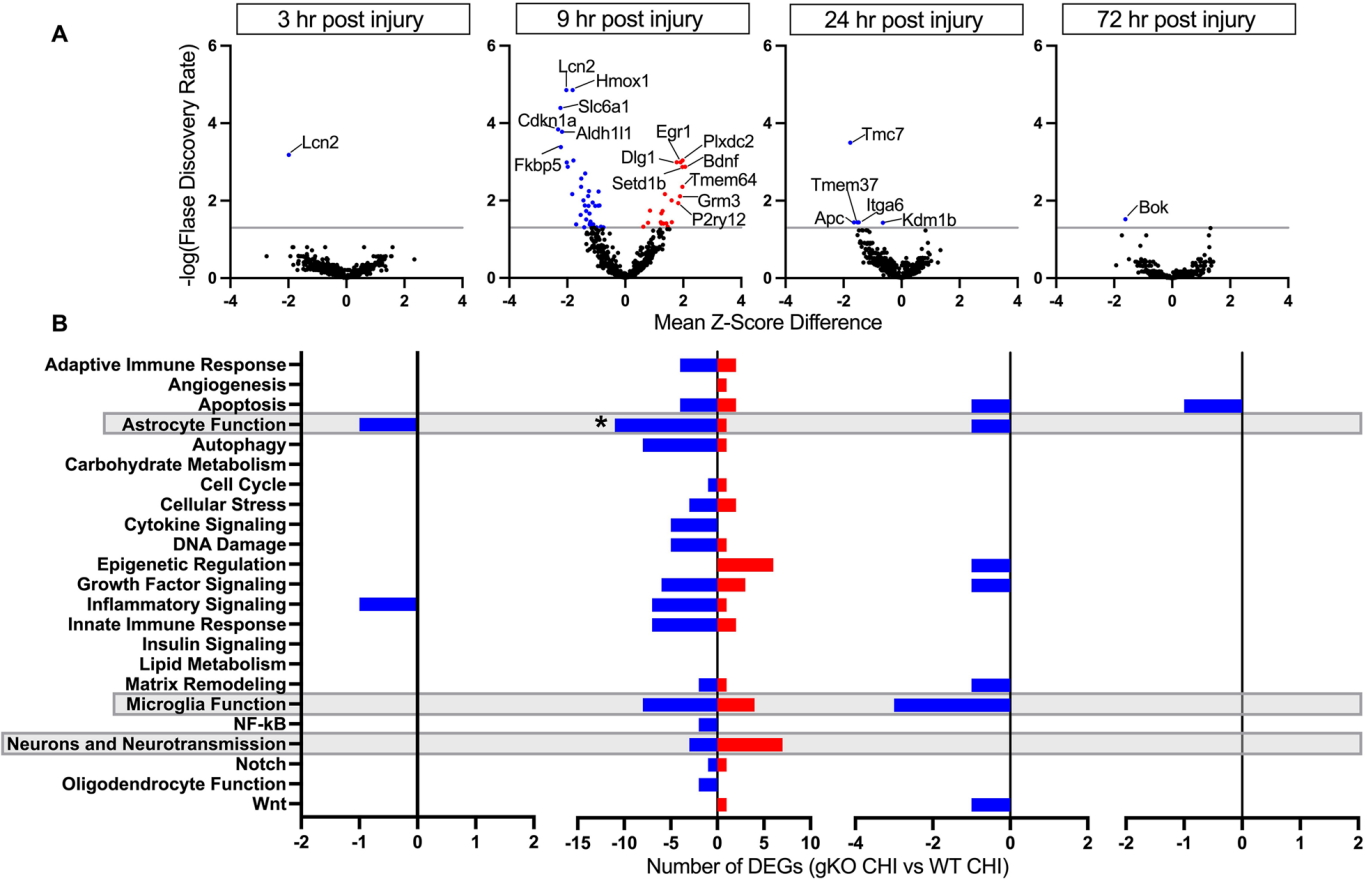
Genotype effect on differentially expressed genes and pathway enrichment analysis in WT and IL-1R1 gKO CHI mice. Direct comparison between IL-1R1 gKO CHI vs WT CHI groups demonstrates IL-1R1 signaling pathway’s temporal specificity in regulating neuroinflammatory gene expression following CHI. NanoString analysis using a mouse Neuroinflammatory panel was used to assess the genotype effect on cortical transcriptional patterns comparing IL-1R1 gKO CHI to WT CHI mice at 3 h post-injury (IL-1R1 gKO CHI n = 5, WT CHI *n* = 6), 9 h post-injury (IL-1R1 gKO CHI *n* = 10, WT CHI n = 11), 24 h post-injury (IL-1R1 gKO CHI *n* = 7, WT CHI *n* = 6), and 72 h post-injury (IL-1R1 gKO CHI n = 7, WT CHI *n* = 6) in 4-month-old mice. **A** Volcano plots of differential gene expression between IL-1R1 gKO CHI vs WT CHI at 3 h, 9 h, 24 h, and 72 h post-injury. Data points above the reference line (grey) represent statistical significance (FDR < 0.05). Positive Mean Z-Score Difference (red dots) indicates higher gene expression in IL-1R1 gKO CHI relative to WT CHI. Negative Mean Z-Score Difference (blue dots) indicates lesser gene expression in IL-1R1 gKO CHI relative to WT CHI. Numbers in the top left corner of each graph indicate the total number of downregulated (blue) and upregulated (red) differentially expressed genes. **B** NanoString pathway analysis comparing the significant DEGs between IL-1R1 gKO CHI vs WT CHI groups. Asterisks (*) indicate that the total number of significant differentially expressed genes (both upregulated and downregulated) within that designated functional category (y-axis) are significant (*p* < 0.05) relative to chance

To elucidate direct transcriptional changes associated with IL-1R1 signaling following CHI, we assessed the effect of the different genotypes by comparing the IL-1R1 gKO + CHI group to the WT + CHI group (Fig. 5A).

At 3 h post-injury, only 1 significant DEG was identified and shown to be downregulated in the IL-1R1 gKO group, lipocalin-2 (*Lcn2*; FDR < 0.001*)*. This comparison suggests that IL-1R1 signaling plays a significant role in *Lcn2* upregulation following CHI. *Lcn2* is a strong marker for reactive astrocytes and can promote inflammatory responses when released from astrocytes [38]. *Lcn2* expression is often upregulated in cerebrovascular diseases, and deletion of LCN2 has frequently been shown to protect against infiltration of inflammatory mediators [39, 40].

At 9 h post-injury (Fig. 5A), we found 58 significant DEGs when comparing IL-1R1 gKO + CHI to WT + CHI. The most significantly downregulated of these DEGs being *Lcn2* (FDR = 0.000014), Heme Oxygenase 1 (*Hmox1*; FDR = 0.000014), and Solute Carrier Family 6 Member 1 (*Slc6a1*; FDR = 0.000040). However, the most downregulated significant DEG was found to be Cyclin Dependent Kinase Inhibitor 1A (*Cdkn1a*; Mean Z-score Difference = − 2.32; FDR = 0.00014). The most significantly upregulated DEGs were Plexin Domain Containing 2 (*Plxdc2*; FDR = 0.00093), Early Growth Response 1 (*Egr1*; FDR = 0.001), and Discs Large MAGUK Scaffold Protein 1 (*Dlg1*; FDR = 0.001). However, the most upregulated significant DEG was found to be Brain Derived Neurotrophic Factor (*Bdnf*; Mean Z-score difference = 2.07; FDR = 0.0013).

At 24 h post-injury (Fig. 5A), we observed 5 significant DEGs, including Transmembrane Channel Like 7 (*Tmc7*; FDR = 0.00032), APC Regulator Of WNT Signaling Pathway (*Apc*; FDR = 0.036), Transmembrane Protein 37 (*Tmem37*; FDR = 0.036), Integrin Subunit Alpha 6 (*Itga6*; FDR = 0.036), and Lysine Demethylase 1B (*Kdm1b*; FDR = 0.037). The most downregulated of these being *Tmc7* with a mean *Z*-score difference of − 1.77. At 72 h post-injury, the only significant DEG found was BCL2 Family Apoptosis Regulator BOK (*Bok*; FDR = 0.030), which we observed to be downregulated.

**Fig. 6:**
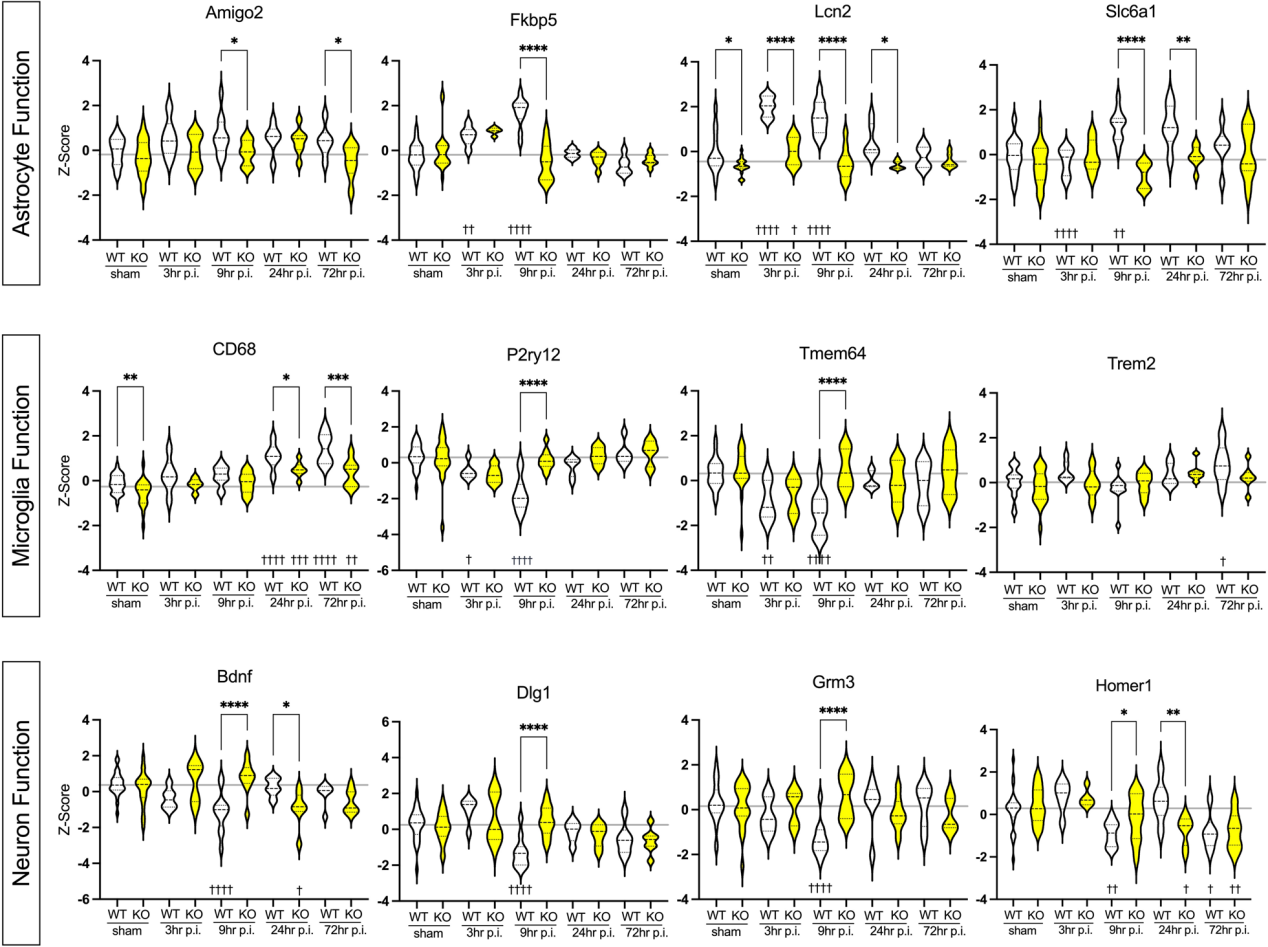
IL-1’s effect on select genes from Astrocyte, Microglia, and Neuron/Neurotransmission functional pathways at 3 h, 9 h, 24 h, and 72 h following a CHI in mice. The first (top) row displays four genes correlated with astrocyte function (*Amigo2, Fkbp5, Lcn2,* and *Slc6a1*). The second (middle) row highlights four genes connected with microglia function (*CD68, P2ry12, Tmem64,* and *Trem2*). The third (bottom) row shows four genes associated with neuron and neurotransmission function (*Bdnf, Dlg1, Grm3,* and *Homer1*). White groups indicate wild-type genotype, whereas yellow groups indicate IL-1R1 gKO genotype. Sham groups are combined of each respective genotype sham (WT sham or IL-1R1 gKO sham) at 3-, 9-, 24-, and 72-h post-sham procedure. Y-axis displays Z-score data for gene expression. X-axis indicates genotype and injury groups. Grey line indicates the reference line of the mean Z-score for combined WT and IL-1R1 gKO sham groups. To compare genes with significant effects in the two-way ANOVA (Table 1), post hoc tests were applied. A Dunnett's multiple comparison test was used to compare 3 h, 9 h, 24 h, and 72 h to the sham control group (^†^*p* < 0.05, ^††^*p* < 0.01, ^†††^*p* = 0.001, ^††††^*p* = 0.0001). The effect of genotype at each time point was compared using an unpaired two-tailed *t*-test (*p < 0.05, **p < 0.01, ***p = 0.001, ****p =  < 0.0001)

Prior research has demonstrated the expression of IL-1R1 on various cell types, including microglia, astrocytes, and neurons, among others [16]. To further visualize the temporal pattern of gene changes, we focused on genes from the pathway enrichment analysis associated with these cell types, which are included in the NanoString Neuroinflammatory panel. As shown in Fig. 6 and Table 1, we present a representative subset of genes from the pathway enrichment analysis.

**Table 1:** Two-way ANOVA of representative genes from pathway enrichment analysis

		Gene	Genotype	Injury	Interaction
Fig. 6	Astrocyte function	*Amigo2*	0.0013**	0.0209*	0.4594
		*Fkbp5*	0.0125*	< 0.001***	< 0.001***
		*Lcn2*	< 0.001***	< 0.001***	< 0.001***
		*Slc6a1*	< 0.001***	0.0122*	< 0.001***
	Microglia function	*Cd68*	< 0.001***	< 0.001***	0.2352
		*P2ry12*	0.0108*	< 0.001***	< 0.001***
		*Tmem64*	0.0024**	< 0.001***	0.0006***
		*Trem2*	0.1414	0.0036**	0.2985
	Neuron function	*Bdnf*	0.2514	0.0203*	< 0.001***
		*Dlg1*	0.3804	< 0.001***	0.0003***
		*Grm3*	0.1599	0.1842	0.0002***
		*Homer1*	0.6559	< 0.001***	0.0136*
Fig. 7	Chemokine	*Ccl2*	0.0197*	< 0.001***	0.0003***
		*Ccl3*	0.023*	< 0.001***	0.0057**
		*Ccl4*	0.0419*	< 0.001***	0.0002***
		*Tnf*	0.9953	0.0983	0.799

∗*p* < 0.05, ∗∗*p* < 0.01, ∗∗∗*p* < 0.001

### IL-1R1 signaling significantly affects gene expression related to astrocyte function at 9 h post-CHI

After characterizing the genotype effect on gene expression following a CHI, highlighted in Fig. 5A, we sought to assess these effects within the functional pathways associated with the identified significant DEGs by performing another pathway enrichment analysis (Fig. 5B). Interestingly, we showed that the genotype effect comparing IL-1R1 gKO + CHI to WT + CHI resulted in a significant effect on gene expression in the astrocyte functional pathway (*p* = 0.0005) at 9 h post-injury. Within the 757 genes investigated with the NanoString Neuroinflammatory panel, 55 of the genes are associated with the astrocyte function pathway. Of these 55 genes, 22% of them were found to be significantly differentially expressed, with 11 of these DEGs being downregulated in the IL-1R1 gKO + CHI group, and 1 DEG being upregulated in the IL-1R1 gKO + CHI group when compared to the WT + CHI group. The sole upregulated significant DEG was found to be Glutamate Metabotropic Receptor 3 (*Grm3*; Mean Z-score difference = 1.97; FDR = 0.0044). This comparison suggests that IL-1R1 activation leads to the downregulation of *Grm3* post-CHI. The Glutamate Metabotropic Receptor 3 (mGluR3) is a G-protein coupled receptor (GPCR) that plays a vital role in modulating synaptic transmission, synaptic plasticity, and neuronal excitability [41]. Its inhibitory nature enables mGluR3 to influence neuronal injury and provide neuroprotection against various insults [42].

Of the 11 significant downregulated DEGs, the most downregulated were found to be *Slc6a1* (Mean *Z*-score difference = − 2.23; FDR = 0.00004), FKBP Prolyl Isomerase 5 (*Fkbp5*; Mean *Z*-score difference = − 2.22; FDR = 0.00042), and Aldehyde Dehydrogenase 1 Family Member L1 (*Aldh1l1*; Mean *Z*-score difference = − 2.18; FDR = 0.00017). This suggests that IL-1R1 signaling leads to the upregulation of these genes following CHI. *SLC6A1* encodes the GABA transporter 1 (GAT-1) protein, which plays a critical role in neurotransmission and is responsible for the reuptake of GABA into presynaptic neurons and astrocytes. Human variants of *SLC6A1*, associated with partial or complete loss-of-function of GAT-1, are linked to a range of neurodevelopmental disorders, including epilepsy [43]. FKBP5, also known as FK506-binding protein 5, is a co-chaperone protein that modulates the activity of the glucocorticoid receptor (GR). It has been associated with increased susceptibility to stress, including neuronal injury and neuroinflammation [44].

### IL-1R1 signaling regulates gene expression of prominent chemokines

**Fig. 7:**
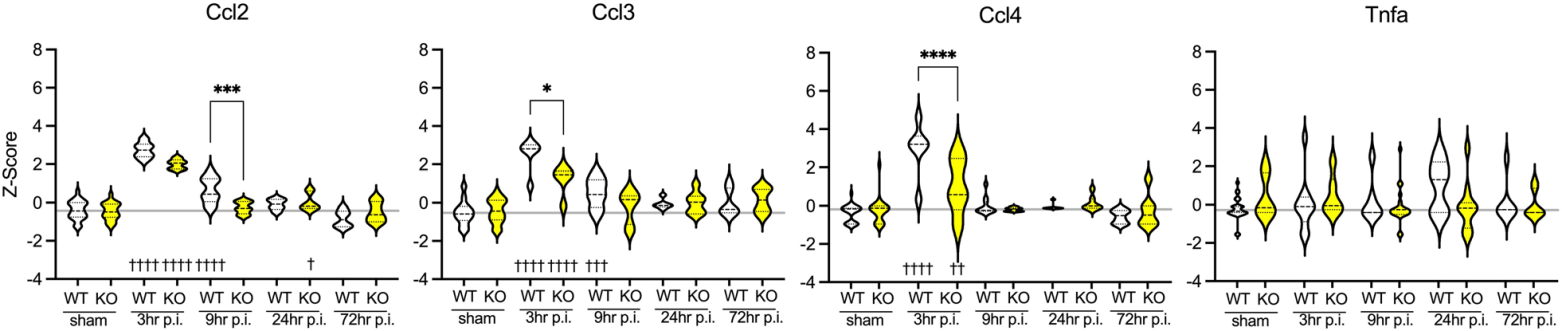
IL-1’s effect on chemokines at 3 h, 9 h, 24 h, and 72 h following a CHI in mice. The chemokine genes assessed include *Ccl2, Ccl3, Ccl4*. White groups indicate wild-type genotype. Yellow groups indicate IL-1R1 gKO genotype. Sham groups are a conglomeration of each respective genotype sham (WT sham or IL-1R1 gKO sham) at 3-, 9-, 24-, and 72-h post-sham procedure. Y-axis displays Z-score data for gene expression. X-axis indicates genotype and injury groups. Grey line indicates the reference line of the mean Z-score for combined WT and IL-1R1 gKO sham groups. To compare genes with significant effects in the two-way ANOVA (Table 1), post hoc tests were applied. A Dunnett's multiple comparison test was used to compare 3 h, 9 h, 24 h, and 72 h to the sham control group (^†^*p* < 0.05, ^††^*p* < 0.01, ^†††^*p* = 0.001, ^††††^*p* = 0.0001). The effect of genotype at each time point was compared using an unpaired two-tailed *t*-test (*p < 0.05, **p < 0.01, ***p = 0.001, ****p =< 0.0001)

Although chemokines were not part of a specific NanoString pathway, several chemokines were identified as the most significantly differentially expressed genes in previous analyses. As a result, in order to investigate the IL-1 specificity in regulating gene expression for selected chemokines, we directly compared the changes in gene expression between WT and IL-1R1 gKO mice at 3 h, 9 h, 24 h, and 72 h following CHI (Fig. 7 and Table 1). Of note, IL-1 signaling exhibited specificity in regulated *Ccl3* (*p* < 0.05) and *Ccl4* (*p* < 0.0001) 3 h post-injury, with gene expression levels returning to baseline at 9 h post-injury and so on. IL-1 also showed specificity in its effect on *Ccl2* gene expression levels at 9 h post-injury (*p* < 0.001). This further emphasizes the temporal role IL-1 signaling may have in potentiating secondary inflammatory signaling molecules in the early-acute phase post-injury. Interestingly, IL-1 signaling did not affect the propagation of *Tnfa* gene expression.

## Discussion

**Fig. 8:**
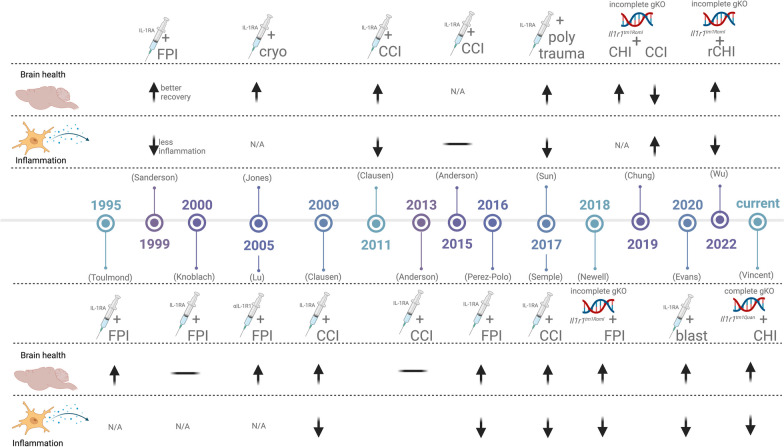
Summary of prior studies evaluating the function of IL-1 / IL-1R1 signaling in experimental models of TBI. This figure provides a chronological summary of 14 studies published between 1995 and 2022 that evaluated the role of interleukin-1 (IL-1) signaling in traumatic brain injury (TBI) using rodent models, including Toulmond and Rothwell (1995), Sanderson et al. (1999), Knoblach and Faden (2000), Jones et al. (2005), Lu et al. (2005), Clausen et al. (2009), Clausen et al. (2011), Anderson et al. (2013), Anderson et al. (2015), Perez-Polo et al. (2016), Sun et al. (2017), Semple et al. (2017), Newell et al. (2018), Chung et al. (2019), Evans et al. (2020), Wu et al. (2022) [19–34]. The symbols used in the figure are as follows: up arrow denotes improvement in brain health or decreased neuroinflammation with IL-1/IL-1R1 targeted intervention compared to control, down arrow denotes worsening in brain health or increased neuroinflammation with IL-1/IL-1R1 targeted intervention compared to control, and a horizontal line denotes no change. These studies collectively demonstrated the involvement of IL-1 signaling in TBI-induced brain health outcomes, such as neuronal loss and behavioral phenotypes, and neuroinflammation outcomes, such as measurements of reactive microglia or astrocytes, immune cell infiltration and cytokine levels. The abbreviations used in the figure are CCI (controlled cortical impact), FPI (fluid percussion injury), and CHI (closed-head injury)

Cytokines, including IL-1, are extensively measured in human and animal models as inflammation biomarkers [7, 45, 46]. A wealth of evidence indicates that IL-1 levels are elevated centrally and systemically after a brain injury, including mild TBI [13, 46, 47]. However, numerous assumptions are made regarding cytokine function in the central nervous system (CNS). These assumptions, such as their involvement in signal transduction pathways, functioning as mitogens, or inducing cell death, are largely based on extrapolating their known systemic immunological roles [9, 48]. Yet, cytokines exhibit unique, non-immunological functions when interacting with receptors on neural cells, such as modulating synaptic plasticity, which can have can have both beneficial and detrimental effects on synaptic plasticity, depending on the context and the specific cytokine involved [49, 50]. Although numerous assumptions have been made about cytokine function in the central nervous system, almost three decades of research in multiple animal models of TBI have revealed that IL-1R1 signaling is involved in several aspects of the neurological sequelae, such as increased inflammation, cognitive impairment, and increased seizure susceptibility following TBI (Fig. 8) [19–34]. Despite these successful studies, comprehensive testing of assumptions regarding the basic physiological function of IL-1 after an injury is needed to understand the relevance of inflammatory cytokines as biomarkers of injury and to advance inflammatory and immunomodulatory treatments for neurotrauma.

Intending to expand our understanding of IL-1's unique functions in the CNS and its role in TBI, we delved into the role of IL-1R1 signaling in chronic behavioral impairment and neuroinflammation after brain injury. IL-1 is a proinflammatory cytokine that contributes to inflammation in various diseases and following injury by activating its primary cell-surface receptor, IL-1R1. While the release of cytokines, including IL-1, is a well-established cause of inflammation following tissue injury, the extent of IL-1R1 signaling in regulating neuroinflammation after brain injury is still being explored. This study established that IL-1R1 signaling plays a role in chronic behavioral impairment following a single mild closed-head injury (mCHI) 14 weeks post-injury. Furthermore, we demonstrated that the loss of IL-1R1 signaling alleviates this behavioral impairment and reduces the reactive microglial burden in the neocortex and the reactive astrocyte burden in the corpus callosum and hippocampus. “Our findings after CHI using an IL-1R1 KO mouse model with no IL-1-induced CNS effects agree with prior studies using an incomplete IL-1R1 KO mouse model (Fig. 8), which is which expresses an IL-1 receptor in the CNS, which activates IL-1-dependent signaling when bound to the alternative IL-1 receptor accessory protein, called IL-1RAcPb [18]. These findings highlight that after a CHI, the canonical IL-1R1/IL-1RAcP pathway is the dominant driver of inflammation and likely cognitive impairment. Additionally, these studies highlight the need for a better understanding of IL-1R/IL-1RAcPb. Together with the findings of other studies, our work supports the notion that IL-1/IL-1R1 is more than a simple biomarker of injury and may contribute to worsened outcomes or impaired recovery.

We hypothesized that IL-1's impact might be greatest during the acute phase after injury, when IL-1 levels are the highest (Fig. 2A). Thus, we designed an experiment to test the role of IL-1R1 signaling at this pivotal time. To this end, we examined cortical gene expression during the acute neuroinflammatory process following a CHI in mice. We broadly characterized neuroinflammatory gene changes within the functional pathways in the neocortex using a nanostring assay on bulk cortical RNA. We found that IL-1R1 signaling has a specific temporal effect on cortical gene expression following a CHI in mice. Specifically, our results demonstrate that IL-1R1 signaling predominantly affects the transcriptomic profile 9 h post-injury in WT mice, which correlates with previous work showing peak expression of IL-1β 9 h post-CHI [13]. Furthermore, global deletion of the *Il1r1* gene significantly affected the expression of genes related to astrocyte function following CHI. Moreover, the deletion of the *Il1r1* gene not only reduced inflammation following CHI, but also diminished the expression of commonly upregulated chemokines post-injury, such as Ccl2, Ccl3, Ccl4, and Ccl7.

Considering the importance of post-traumatic inflammation for debris removal and tissue repair and the potential risks associated with suppressing inflammation, such as increased infections, it is crucial to recognize that interventions targeting inflammation may not always lead to brain protection and faster recovery. Indeed, previous studies have shown that the loss of IL-1R1 can result in worse outcomes following a controlled cortical impact model of TBI [32], indicating that injury severity and biomechanics can lead to altered cellular and temporal contributions of inflammation to TBI sequelae. This highlights the need to investigate the temporal dynamics of the inflammatory response since the intricate nature of brain injuries necessitates different interventions at distinct stages after the injury or injury biomarkers.

Our data suggest that the elevation of IL-1 levels primarily drives the acute inflammatory response within the first 24 h post-injury. The inflammatory transcriptomic profile demonstrates that IL-1R1 at 9 h post-injury is responsible for almost all the inflammatory changes observed in WT mice after injury, as measured by this assay. However, this effect is limited to when the brain's IL-1β levels are elevated following a mild injury. These findings reveal a potentially narrow therapeutic window in this mouse model, occurring between 3 and 12 h post-injury, which might be the optimal period for targeting IL-1 signaling to achieve a neuroprotective effect during the acute inflammatory response after injury. Consequently, we hypothesize that an intervention targeting IL-1/IL-1R1 should be administered within this narrow therapeutic window, likely around 3–9 h post-injury, to maximize the reduction of inflammation. This hypothesis aligns with our previous research using a brain-penetrating small molecule inhibitor that suppresses IL-1 β production, where we observed that administering the inhibitor at 1 and 3 h post-injury effectively alleviated the cognitive deficits in the model [13].

A limitation of our current study is that the IL-1R1 knockout is constitutive. Consequently, we cannot distinguish between the acute and chronic effects of IL-1R1 signaling on the behavioral outcomes we measured. The effects we observed may result from suppressing the acute inflammatory response, as described above. Still, detrimental IL-1R1 signaling could also occur during the chronic phase after injury, acting in ways other than increasing inflammation. For instance, neurons express the IL-1R1 receptor. The literature has shown that blocking IL-1 signaling can lead to learning and memory impairments and dysfunction in synaptic plasticity [51]. Activation of neuronal IL-1R1 signaling is important for aspects of synaptic plasticity, including the development of hyper- and hypo-responsive synapses. Previous studies have demonstrated a potential role for IL-1R1 in apparent synaptic plasticity and enhanced post-traumatic seizure susceptibility [30]. IL-1R1 is also involved in allodynia and hyperalgesia [52, 53]. However, whether IL-1R1 is involved in post-concussive symptoms such as headaches is currently unknown. Thus, future studies that manipulate IL-1R1 signaling outside the acute inflammatory wave to determine if there would be any benefit in targeting IL-1/IL-1R after the chronic phase of the injury are needed. Additional studies are also needed to evaluate the IL-1R1-dependent protein factors that may mediate signaling between cells.

The current study was conducted on naïve mice. However, recent work has demonstrated that repetitive immune challenges can alter the cell-type expression of the IL-1R1 receptor [54, 55]. This raises the possibility that IL-1 may exert different biological effects depending on initial inflammatory conditions, such as injury, infections, or age. Indeed, previous work has shown that aging alters the neuronal IL-1R1 signaling complex to enhance classical inflammatory signal transduction cascades [56–58]. However, we do not know if repetitive TBI has a similar effect. Nonetheless, if the IL-1R1 signaling complex is primed following each inflammatory insult, this may underscore the need to more aggressively manage IL-1/IL-1R1 following repetitive insults or in older adults than younger adults. 

## Conclusions

This study provides further insight into IL-1R1 function in amplifying the neuroinflammatory cascade following CHI in mice and demonstrates that suppression of IL-1R1 signaling offers long-term protective effects on brain health that is likely independent of its transient effects on inflammatory gene expression in the acute period, as IL-1R1 appears to be an early but transient mediator of post-injury gene inflammatory expression, particularly during the 9 h post-injury timeframe. The affects in rescuing behavior deficits and reducing microglia and astrocyte reactivity at 14 weeks post-CHI, will require future studies that can establish additional, potentially cell-type specific functions of IL-1R1 during the chronic phase after injury, or link how the acute changes in the neuroinflammatory cascade have lasting effect on chronic inflammation and neuronal physiology, which may provide valuable insights into IL-1 function in long-term consequences of TBI.

### Supplementary Information

**Additional file 1: Fig. S1.** Pre-operative body weight and post-operative righting reflex time in WT and IL-1R1 gKO mice.

**Additional file 2: Table S1.** All z-scores and log2-transformed values for NanoString data, with raw data for active avoidance test and immunohistochemical staining.

## Data Availability

All data generated or analyzed during this study are included in this published article and its supplementary information files (Additional file 2).

## Abbreviations

ANOVA: Analysis of variance
C4a: Complement component 4A
CD68: Cluster of differentiation 68
CDKN1A: Cyclin dependent kinase inhibitor 1A
CHI: Closed head injury
Clecl7a: C-type lectin domain family 7 member A
Ccl: Chemokine (C-C motif) Ligand
CNS: Central nervous system
CS: Conditioned stimulus
DAB: 3,3'-Diaminobenzidine tetra-hydrochloride
DAMPs: Damage-associated molecular patterns
DEGs: Differentially expressed genes
DLG1: Discs large homolog 1
EGR1: Early growth response factor 1
FDR: False discovery rate
FKBP5: FKBP prolyl isomerase 5
GFAP: Glial fibrillary acidic protein
gKO: Global knockout
IBA1: Ionized calcium binding adapter molecule 1
IHC: Immunohistochemical
IL-1: Interleukin-1
IL-1β: Interleukin-1 beta
IL-1R1: Interleukin-1 receptor 1
IL-1R1 gKO: Interleukin-1 receptor type 1 global knockout
ITI: Intertrial interval
KO: Knockout
mTBI: Mild traumatic brain injury
Nfkβ: Nuclear factor kappa beta
NGS: Normal goat serum
PBS: Phosphate-buffered saline
P2RY12: Purinergic receptor P2Y12
Pole: DNA polymerase Epsilon
Ptgs2: Prostaglandin-endoperoxide synthase 2
RIN: RNA integrity number
SESN1: Sestrin 1
Stmn1: Stathmin 1
TBI: Traumatic brain injury
TIMP1: Tissue inhibitor of metalloproteinases 1
Tnfα: Tumor necrosis factor alpha
US: Unconditioned stimulus
WT: Wild-type
