# Correction : IL-1R1 signaling in TBI: assessing chronic impacts and neuroinflammatory dynamics in a mouse model of mild closed-head injury

**DOI:** 10.1186/s12974-023-02967-8

**Published:** 2023-11-30

**Authors:** Jonathan C. Vincent, Colleen N. Garnett, James B. Watson, Emma K. Higgins, Teresa Macheda, Lydia Sanders, Kelly N. Roberts, Ryan K. Shahidehpour, Eric M. Blalock, Ning Quan, Adam D. Bachstetter

**Affiliations:** 1 Department of Neuroscience, University of Kentucky, 741 S. Limestone St., Lexington, KY 40536 USA; 2 Spinal Cord and Brain Injury Research Center, University of Kentucky, Lexington, KY USA; 3 Sanders‑Brown Center on Aging, University of Kentucky, Lexington, KY USA; 4 MD/PhD Program, University of Kentucky, Lexington, KY USA; 5 Department of Cell, Developmental, and Integrative Biology, University of Alabama at Birmingham, Birmingham, AL USA; 6 Department of Pharmacology and Nutritional Sciences, University of Kentucky, Lexington, KY USA; 7 Department of Biomedical Science, Charles E. Schmidt College of Medicine and Brain Institute, Florida Atlantic University, Jupiter, FL USA

**Correction** **:** **Journal of Neuroinflammation (2023) 20:248** **https://doi.org/10.1186/s12974-023-02934-3**

In this article [1], the author name Ning Quan was incorrectly written as Ning Qaun.

The original article has been corrected.
