# Supplementary material for: IL-1R1 signaling in TBI: assessing chronic impacts and neuroinflammatory dynamics in a mouse model of mild closed-head injury
Source: J Neuroinflammation. 2023 Oct 26;20:248. doi: 10.1186/s12974-023-02934-3 (PMC10601112; doi:10.1186/s12974-023-02934-3)
Supplement: Supplementary file 1 — Additional file 1: Fig. S1. Pre-operative body weight and post-operative righting reflex time in WT and IL-1R1 gKO mice. [file 12974_2023_2934_MOESM1_ESM.docx]

**IL-1R1 signaling in TBI: Assessing chronic impacts and neuroinflammatory dynamics in a mouse model of mild closed-head injury.**

Jonathan C. Vincent^1,2,3,4^, Colleen N. Garnett^1,2,3,5^, James B. Watson^1,2^, Emma K. Higgins^1,2^, Teresa Macheda^1,2^, Lydia Sanders^1,2^, Kelly N. Roberts^1,2^, Ryan K. Shahidehpour^1,2,3^, Eric M. Blalock^6^, Ning Qaun^7^, Adam D. Bachstetter^1,2,3^

^1^Department of Neuroscience, University of Kentucky, Lexington, KY, United States

^2^Spinal Cord and Brain Injury Research Center, University of Kentucky, Lexington, KY, United States

^3^Sanders-Brown Center on Aging, University of Kentucky, Lexington, KY, United States

^4^MD/PhD Program, University of Kentucky, Lexington, KY, United States

^5^Department of Cell, Developmental, and Integrative Biology, University of Alabama at Birmingham, Birmingham, AL, United States

^6^Department of Pharmacology and Nutritional Sciences, University of Kentucky, Lexington, KY, United States

^7^Department of Biomedical Science, Charles E. Schmidt College of Medicine and Brain Institute, Florida Atlantic University, Jupiter, FL, United States

**CONFLICTS OF INTEREST**

The authors declare that there are no commercial, financial, or other relationships that could be construed as a potential conflict of interest when working on this research.

**CORRESPODING AUTHOR:**

Adam D. Bachstetter

741 S. Limestone St.

Lexington, KY 40536

Phone: 859-218-4315

Email: adam.bachstetter@uky.edu


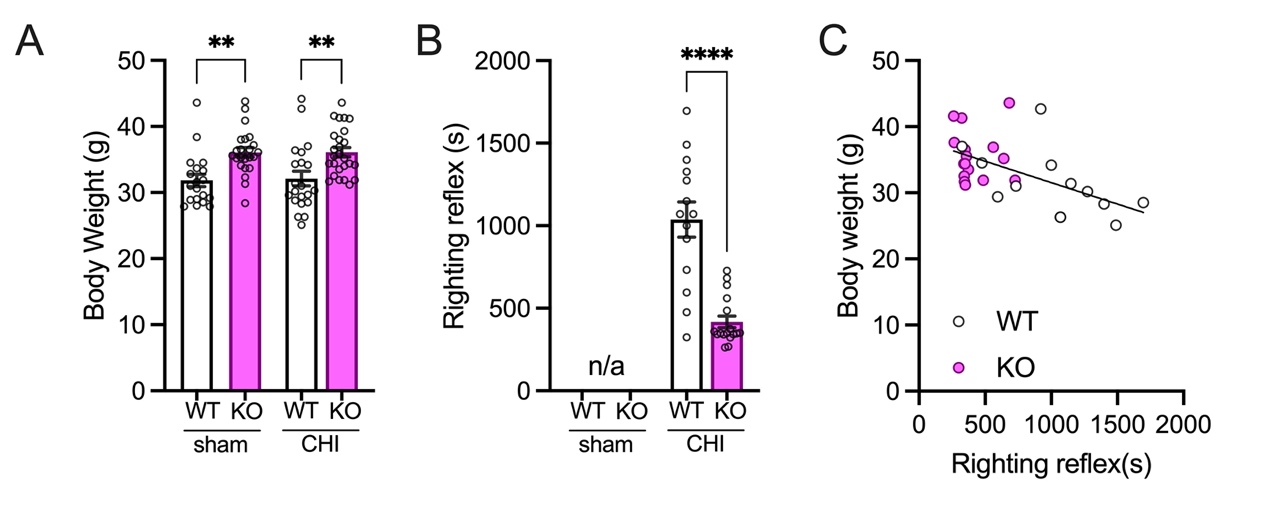


**Supplemental Figure 1. Pre-operative body weight and post-operative righting reflex time in WT and IL-1R1 gKO mice. (A)** Interleukin-1 receptor 1 global knock-out (KO) mice weighted 12% more than the wild-type (WT). **(B)** The KO mice were righted 40% quicker after the CHI than the WT mice. **(C)** Pre-operative body weight correlated with righting reflex time. **p<0.005, ****p<0.0001.
